# Patient information leaflets (PILs) for UK randomised controlled trials: a feasibility study exploring whether they contain information to support decision making about trial participation

**DOI:** 10.1186/1745-6215-15-62

**Published:** 2014-02-18

**Authors:** Katie Gillies, Wan Huang, Zoë Skea, Jamie Brehaut, Seonaidh Cotton

**Affiliations:** 1 Health Services Research Unit, University of Aberdeen, Health Sciences Building, Foresterhill, Aberdeen AB25 2ZD, UK; 2 Clinical Epidemiology Program, Ottawa Hospital Research Institute, The Ottawa Hospital, General Campus, 501 Smyth Road, Centre for Practice-Changing Research, Box 201B, Ottawa, ON KIH 8L6, Canada

**Keywords:** Patient information leaflets, Informed consent, Randomised controlled trials, Decision support

## Abstract

**Background:**

Informed consent is regarded as a cornerstone of ethical healthcare research and is a requirement for most clinical research studies. Guidelines suggest that prospective randomised controlled trial (RCT) participants should understand a basic amount of key information about the RCTs they are being asked to enrol in in order to provide valid informed consent. This information is usually provided to potential participants in a patient information leaflet (PIL). There is evidence that some trial participants fail to understand key components of trial processes or rationale. As such, the existing approach to information provision for potential RCT participants may not be optimal. Decision aids have been used for a variety of treatment and screening decisions to improve knowledge, but focus more on overall decision quality, and may be helpful to those making decisions about participating in an RCT. We investigated the feasibility of using a tool to identify which items recommended for good quality decision making are present in UK PILs.

**Methods:**

PILs were sampled from UK registered Clinical Trials Unit websites across a range of clinical areas. The evaluation tool, which is based on standards for supporting decision making, was applied to 20 PILs. Two researchers independently rated each PIL using the tool. In addition, word count and readability were assessed.

**Results:**

PILs scored poorly on the evaluation tool with the majority of leaflets scoring less than 50%. Specifically, presenting probabilities, clarifying and expressing values and structured guidance in deliberation and communication sub-sections scored consistently poorly. Tool score was associated with word count (r = 0.802, *P* <0.01); there was no association between score and readability (r = -0.372, *P* = 0.106).

**Conclusions:**

The tool was feasible to use to evaluate PILs for UK RCTs. PILs did not meet current standards of information to support good quality decision making. Writers of information leaflets could use the evaluation tool as a framework during PIL development to help ensure that items are included which promote and support more informed decisions about trial participation. Further research is required to evaluate the inclusion of such information.

## Background

Informed consent forms the cornerstone of ethical requirements in healthcare research [1,2]. In the context of healthcare research, the giving of informed consent signifies that an individual has made an informed and voluntary decision about their participation in a research study. Randomised controlled trials (RCTs) usually require all participants to sign an informed consent document indicating that they have understood the information they have been provided with before they commence participation in the trial [1].

To assist in making an informed decision about participation, potential trial participants in the UK are provided with a patient information leaflet (PIL) that contains information about the RCT. The information included in PILs is guided by the Declaration of Helsinki, the International Conference on Harmonisation Good Clinical Practice (ICH GCP) and also by national guidance such as the National Research Ethics Service (NRES) [1,3,4]. As defined by the guidance, the PIL should include, at minimum, information about: the purpose of the trial; procedures; interventions; possible risks and benefits; sources of finance; potential conflicts of interest; and the researcher’s affiliation [1,3]. The guidance for informational requirements covers predominantly fact-based information and is standardised at a population level [1,3].

Currently the PIL and consent form are the only components of the consent decision making process for RCTs that are formally regulated, through specific guidance, and reviewed by ethics committees or internal review bodies [1]. However, as with decisions about treatment, potential participants vary in the amount of information they desire when faced with a decision about trial participation [5-7]. As such, within a trial context where the information minimum has been pre-determined by the guidance [1,3,4], many potential participants’ preferences for information may be exceeded [6] and other approaches to supporting decision making may be required. Despite this, there is evidence that some trial participants, both those considering participation and those actively enrolled in clinical trials, fail to understand key components of the trial processes or rationale [8-10]. This has ranged from misunderstandings about: risks [11]; the right to withdraw [11]; confidentiality [12]; side effects [13]; and purpose of the trial [14]. This suggests that in the context of trial participation, existing approaches to information provision may be sub-optimal and some decisions may not have been based on a full understanding or consideration of all the relevant issues. This may be because the information is too complex or not designed to support an informed decision but rather to present factual information to the potential participant. A range of studies have explored ways of improving information, and have tended to focus on the content and structure of the information [8]. For example: the length of the information sheet, that is, short versus long leaflets [10,15]; simplified or enhanced versions of the same PIL [16,17]; patient-specific information versus generic information [18,19]; linguistic analysis of leaflets [20]; consumer involvement in development of PILs [21]; audio-visual information [22]; computer-based information [23]; and user testing to improve content [24].

As discussed above, the literature to date has tended to focus on the provision of information, to improve understanding about trial processes, with the aim of making the consent process more 'informed’. However, informed decision making in the context of trial participation is a complex process and requires more than just greater understanding or comprehension of certain fact-based information. For example, key considerations which are regarded by established theories of decision making as being important for making 'good’ decisions (for example considering the alternatives (for example standard care and what that is), making trade-offs and evaluating potential outcomes of the decision [25]) might not be presented or discussed at all during the informed consent discussion or explicit in the PIL. These omissions may stem from the conventional conceptualisation of trial participation as an act of 'informed consent’. This conceptualisation likely relates to the ethical and regulatory requirements, rather than viewing deliberations and decisions about participation as broader and more complex [26].

Decision aids have been developed for a variety of treatment and screening decisions as a means of improving 'informed decisions’ in particular contexts and across the decision making process [25]. These decision aids provide information about: the available options and any associated outcomes; personalise information by including exercises to help patients think about what matters most to them; and provide ways of communicating this with the healthcare professional to reach a decision [25]. There is substantial evidence to suggest that decision aids can positively influence outcomes, such as: improving knowledge, especially when there is clinical equipoise; providing accurate perceptions of outcome probabilities; and align preferred outcomes with the choice made [25].

As discussed previously, the traditional conceptualisation of informed consent in RCTs is as a behaviour relating to understanding rather than promoting informed decisions. This may have led to the information contained within PILs being focussed on fact-based trial information rather than, for example, what trial participation might mean for that individual. Yet, these personal considerations do play a significant role for people when deciding whether or not to participate in a trial [27] and this is often the type of information contained within treatment decision aids. As such, it is perhaps timely to re-think the way in which decisions about trial participation and discussions about informed consent are supported.

Preliminary studies exploring the potential of decision aids for use in the informed consent process for RCTs have shown promise in that they appear to improve aspects of the decision making process [28-32]. Decision aids to support decisions about trial participation have been shown to be acceptable and valued by potential participants [28,29]. In these studies, the decision aid was a substitute for the existing PIL and contained all of the required information expected to be in a PIL in addition to those expected to be in a decision aid. Specifically, decision aids in this context have been shown to improve understanding about the trial and its associated interventions [28,29], produce low levels of decision conflict [29] and not raise anxiety [28,29]. However, these studies had small sample sizes and were set within a hypothetical trial context. As such, the need for further research has been noted [28-32]. A recent study examined the extent to which existing informed consent documents conform to standards for encouraging good quality decision making as laid out by International Patient Decision Aid Standards (IPDAS) [33,34]. The evaluation tool used in the study by Brehaut and colleagues contained two sections: a series of items derived from the IPDAS and a set of items derived from published guidance on informed consent [34]. Only the first section of the tool, including items developed from the IPDAS, was used and the majority of the included PILs were developed in the US with approximately half being for cancer trials [34]. Guidance for US PILs differs from that for UK PILs with regard to whether specific information is presented in either the information sheet or consent form, with UK consent forms being a one-page document, whereas US consent forms are longer and contain much of the information contained within the UK PIL. Moreover, the study by Brehaut and colleagues did not assess word count or readability, both of which can contribute to understanding of information and length of document.

Previous research has suggested that decision aids for trial participation have the potential to promote good quality decision making [28,29] and specific components of decision aids that have been applied in RCTs have also offered promise [30,35]. Therefore, this study aimed to assess the feasibility of assessing the extent to which items considered important in decision aids are present in existing UK PILs. We used the complete evaluation tool (developed by Brehaut *et al.*[34]), alongside word count and readability 'calculators’, to evaluate PILs across a range of clinical conditions in a UK context.

## Methods

### Sample of patient information leaflets

We screened the websites of 48 UK Clinical Research Collaborations registered (full or provisional) Clinical Trial Units (CTUs) for publicly available PILs from recently completed or on-going RCTs. Eighteen CTUs provided copies of such PILs on their websites. The CTU based in the Institute of the lead researcher (KG) also provided PILs for the study. The total population of publicly available CTU PILs was 60. The following inclusion criteria were applied to the population of 60 PILs: PILs for a RCT that was on-going or completed after 2001 (to coincide with the introduction of the European Clinical Trials Directive 2001/20/EC [36]); PILs designed for a primary RCT, that is, not a follow-on study; and PILs designed for competent adults making a decision about their own participation, not proxy decision makers. PILs that were designed for cluster RCTs, emergency research with retrospective consent and RCTs recruiting children or healthy volunteers were excluded from the study. Subsequently, PILs were sampled purposively, from the identified population of 60, to allow for variation in intervention and CTU. Four main intervention groups were identified: drug; surgical; cognitive; and other (which included physiotherapy studies and smoking cessation trials). A final sample of 26 was purposively selected for: initial evaluation tool feasibility assessment (n = 1); pilot (n = 5); and full analysis (n = 20).

### Evaluation tool

Development of the evaluation tool has been described elsewhere [34]. In brief, the tool is divided into two sections: items derived from the IPDAS (Section A, 32 items) and additional items derived from published guidelines for informed consent (Section B, 27 items) [34]. The two sections are made up of a total of 59 items.

### Data collection

For the purposes of this study, the study team (KG, WH, ZS and SC) carried out an initial evaluation of one PIL to assess whether it was feasible to use the tool to assess PILs from UK RCTs. This was necessary as the tool was developed based on the informational requirements for US and Canadian PILs [34], which differ from UK guidelines with regard to whether specific information is presented in the information sheet or consent form. The team then discussed items for meaning and clarification and developed a coding 'manual’, which contained detailed description of rules for coding, to assist in the analysis (for example, see Additional file 1). In developing the coding manual we opted to rate items using a two-point scale, rather than the four-point scale originally reported. This approach was taken as it is difficult to make a clear distinction between both strongly agree and agree, and strongly disagree and disagree. The two-point scale was defined as follows: 'agree’ (that is, information was present) or 'disagree’ (information was absent), with some items (n = 5) including a 'not applicable’ option. Two researchers (KG and WH) carried out further pilot assessment using the amended scale. They independently rated five PILs using the tool and then compared their scores to identify areas of divergence, which were then discussed by the study team and used to further refine the coding manual. Changes to the coding manual following this pilot stage included clarifying the focus for each question, for example question 1: 'On the first page of the PIL there is a description that potential participants need to make a decision about whether or not to participate in the trial’. The focus for this question was determined as 'potential participants need to make a decision’ and the PIL was required to state this explicitly (for example you need to make a decision about whether or not to participate) rather than implicitly, through wording such as 'you are being invited to participate in a trial’. Further details on items included in the tool and the coding rules associated with them can be seen in Additional file 1. The evaluation scores generated from the five PILs included in the pilot stage are not reported.

The 20 PILs sampled for full analysis were rated independently using the tool by two members of the research team (KG and WH). Evaluation scores for each PIL were recorded and discrepancies were resolved using a third researcher (SC) and the majority decision recorded.

Readability scores were calculated using an online Flesch-Kincaid readability calculator [37]. The Flesch-Kincaid algorithm is based on word length and sentence length, where higher scores indicate material that is easier to read and lower scores indicate material that is harder to read, with scores being presented out of 100 [37]. Word count was measured using the appropriate function in Microsoft Word. Correlation coefficients were used to test the relationship between readability and tool score, and word count and tool score.

### Analysis

Items on the tool were scored as follows: agree = 1 and disagree = 0. Therefore, the maximum evaluation score for a PIL was 59. Where the 'not applicable’ option was selected, the item was not scored and, for the calculation of percentage score, the denominator was reduced by 1. Data from the evaluation tool scores were presented as raw data and as percentages. Higher scores indicate PILs which performed better when applying the evaluation tool.

Inter-rater reliability between the two independent researchers was assessed for the final sample of 20 PILs using unweighted Cohen’s kappa. Pearson correlation coefficients were calculated using IBM SPSS Statistics (IBM Corporation, Armonk, NY, USA), and used to test the relationship between readability and tool score, and word count and tool score. A one-way ANOVA, calculated using IBM SPSS Statistics, was used to test differences in mean group scores between the intervention groups.

## Results

### Patient information leaflets

The 20 PILs included in the final analysis contained five PILs from each of the intervention groups; drug; surgical; cognitive; and other. PILs from all 19 CTUs were included in the final sample and the majority of PILs (16/20) were produced on, or after, 2006 (Additional file 2).

### Evaluation scores

Inter-rater reliability was confirmed with an overall mean kappa score of 0.846. There was variability in total evaluation scores across the sample (Table 1). The highest evaluation score recorded was 38 (64%), while the lowest was 19 (33%). Thirteen (65%) PILs scored a total of 50% or less.

**Table 1:** Scores for patient information leaflets

**Number**	**Overall score (n/59)**	**Score for section A (n/32)**	**Score for section B (n/27)**	**Scores of sub-sections**										
				**Score for sub-sections in section A**						**Score for sub-sections in section B**				
				**Providing information about options in sufficient detail to make a decision (n/12)**	**Presenting probabilities (n/8)**	**Clarifying and expressing values (n/2)**	**Structured guidance in deliberation and communication (n/2)**	**Using evidence (n/4)**	**Disclosure and transparency (n/4)**	**Key elements (n/10)**	**Ethical issues (n/5)**	**Study design (n/5)**	**Formatting (n/6)**	**Style (n/1)**
**Drug interventions**														
01	31 (53%)*	10 (31%)	21 (81%)*	5	0	0	0	1	4	7	4	4	5*	1
02	26 (45%)*	9 (28%)	17 (65%)*	5	0	0	0	1	3	6	3	3	4*	1
03	38 (64%)	13 (41%)	25 (93%)	6	0	1	1	2	3	9	4	5	6	1
04	29 (50%)*	9 (28%)	20 (77%)*	4	0	0	0	1	4	8	3*	3	5	1
05	33 (57%)*	11 (34%)	22 (85%)*	6	0	0	1	1	3	10	3	3	5*	1
**Surgical interventions**														
06	33 (56%)	12 (38%)	21 (78%)	6	0	1	0	1	4	8	4	3	5	1
07	19 (33%)*	7 (22%)	12 (46%)*	2	0	0	1	1	3	3	2	1	5*	1
08	26 (44%)	8 (25%)	18 (67%)	2	0	0	1	1	4	7	3	4	4	0
09	33 (56%)	10 (31%)	23 (85%)	4	0	0	1	1	4	9	3	4	6	1
10	24 (41%)*	6 (19%)	18 (69%)*	3	0	0	0	1	2	5	3	4	5*	1
**Cognitive interventions**														
11	23 (40%)*	6 (19%)	17 (65%)*	2	0	0	0	1	3	8	1	3	4*	1
12	26 (46%)**	7 (22%)	19 (76%)**	1	0	0	1	1	4	8	1*	4	5*	1
13	21 (37%)**	5 (16%)	16 (64%)**	3	0	0	0	0	2	7	0*	3	5*	1
14	23 (40%)*	7 (22%)	16 (62%)*	3	0	0	0	1	3	7	1	3	4*	1
15	28 (48%)*	11 (34%)	17 (65%)*	7	0	0	0	1	3	8	0	3	5*	1
**Other interventions**														
16	24 (42%)**	6 (19%)	18 (72%)**	2	0	0	0	1	3	7	2*	3	5*	1
17	31 (54%)**	10 (31%)	21 (84%)**	5	0	0	0	1	4	9	2*	4	5*	1
18	22 (39%)**	6 (19%)	16 (64%)**	2	0	0	0	1	3	6	2*	2	5*	1
19	30 (53%)**	9 (28%)	21 (84%)**	4	0	0	1	2	2	9	2*	4	5*	1
20	22 (39%)**	5 (16%)	17 (68%)**	2	0	0	0	0	3	7	2*	2	5*	1

*Denominator for overall score, and Section B, reduced by 1 (n = 58 and n = 26, respectively) due to scoring one item as not applicable; **denominator for overall score, and Section B, reduced by 2 (n = 57 and n = 25, respectively) due to scoring two items as not applicable.

The evaluation scores for Section A ranged from 16% to 41%. Evaluation scores for Section B were higher, ranging from 46% to 91%. There were sub-sections within Section A that scored consistently poorly. These were: presenting probabilities; clarifying and expressing values; structured guidance in deliberation and communication; and using evidence. The 'presenting probabilities’ sub-section, which contains eight items, was scored as '0’ across all PILs and as such was the worst performing sub-section.

There was no statistically significant difference (*P* = 0.119) in mean evaluation scores between intervention groups (Table 2). Similarly, there was no statistically significant difference in mean evaluation scores between intervention groups for Section A (*P* = 0.097) or Section B (*P* = 0.209) (Table 2).

**Table 2:** Comparison of intervention group mean scores

**Mean**	**Intervention group**				***P*** **value**
	**Drug**	**Surgical**	**Cognitive**	**Other**	
Overall score (SD)	31 (5)	27 (6)	24 (3)	26 (4)	0.119*
Section A score (SD)	10 (2)	9 (2)	7 (2)	7 (2)	0.097*
Section B score (SD)	21 (4)	18 (3)	17 (1)	19 (2)	0.209*
Readability (SD)	57.8 (5.6)	59.9 (4.0)	60.8 (8.0)	64.5 (7.4)	0.449*
Word count (SD)	2,572 (1,197)	1,896 (979)	1,523 (623)	1,422 (754)	0.224*

*P*, statistical significance of ANOVA test of scores across intervention groups; SD, standard deviation. *Not statistically significant.

### Evaluation score and readability

**Figure 1:**
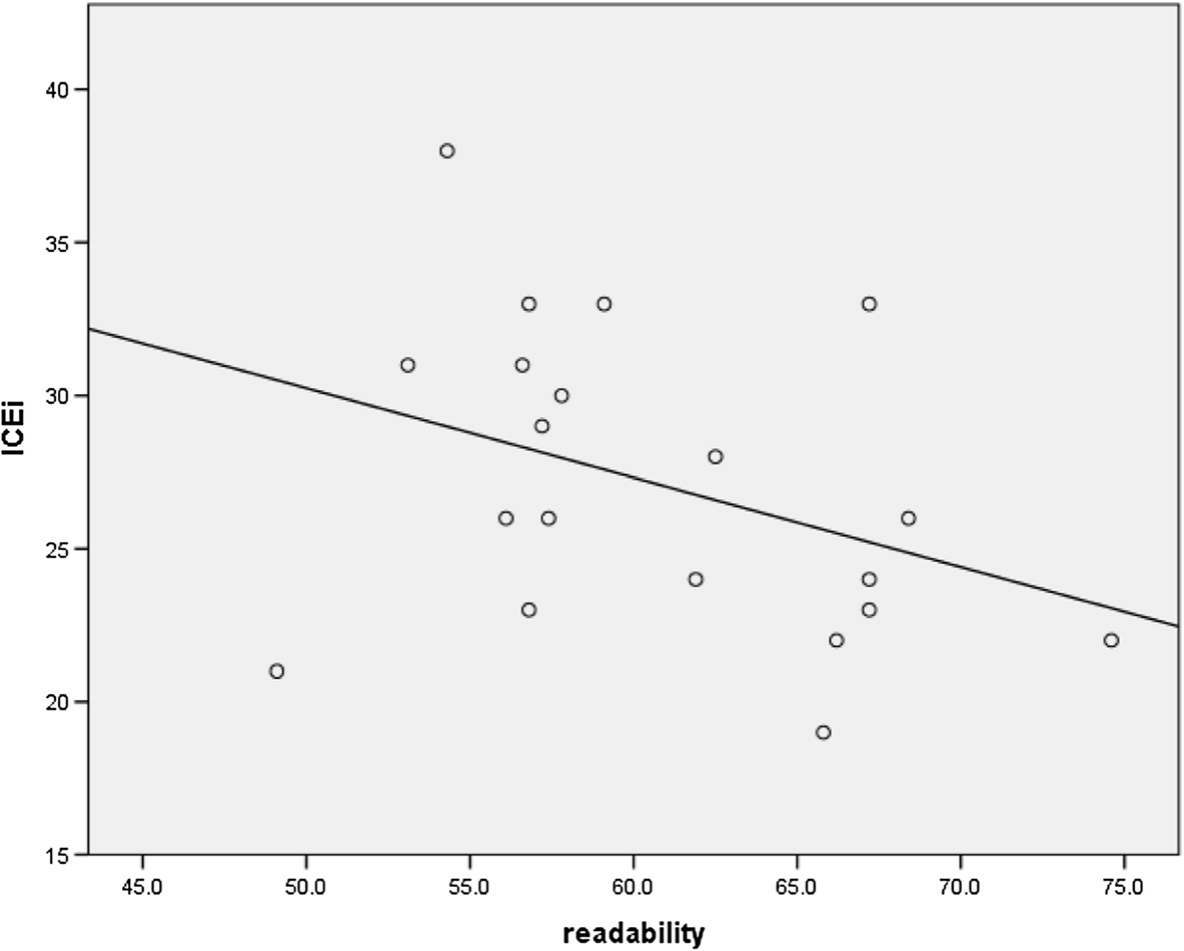
Correlation of readability and overall score.

Readability scores ranged from 49 to 75 with a mean score of 60.8 (SD = 6.4) (scores of 60 to 70 suggest understanding by 13- to 15-year-olds). There were no statistically significant differences in mean readability scores between the intervention groups (*P* = 0.449; Table 2). Readability did not correlate with evaluation score (r = -0.372, *P* = 0.106; Figure 1).

### Evaluation score and word count

**Figure 2:**
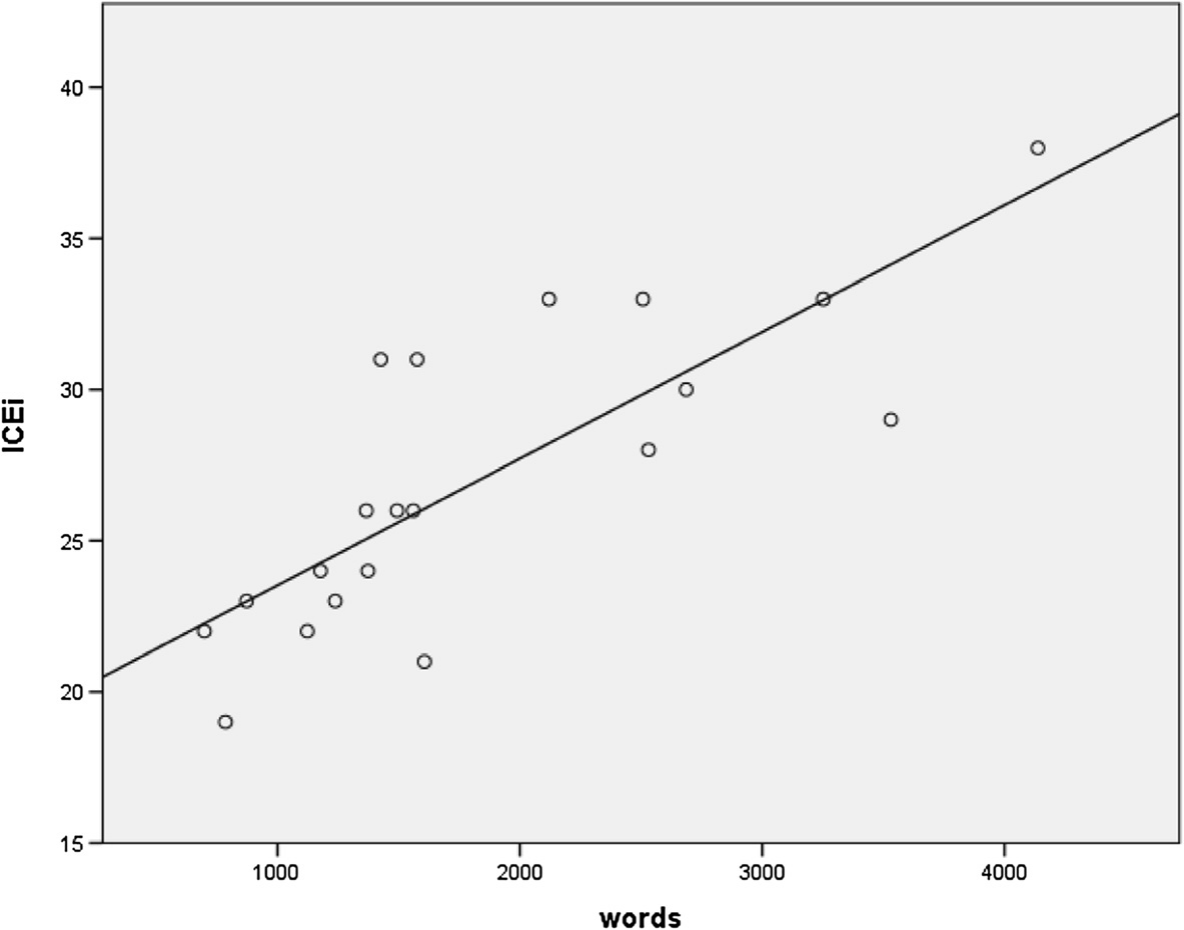
Correlation of word count and overall score.

Word count ranged from 698 to 4,138 words, with a mean word count of 1,853 (SD = 960). There were no statistically significant differences in word count between intervention groups (*P* = 0.224; Table 2). However, unlike readability, word count had a positive correlation with evaluation score and was statistically significant (r = 0.802, *P* <0.01; Figure 2).

## Discussion

This study evaluated PILs from UK RCTs using an evaluation tool based on the IPDAS [33] and informed consent guidelines.

We assumed that all included PILs had been reviewed by an ethics committee and had been given a favourable opinion. Despite this, we found that the majority of PILs did not perform well using the tool (based on a score of less than 50%). All the PILs scored more poorly on Section A as compared to Section B. Our results provide an indication that information shown to support high quality decision making in other contexts is lacking in PILs for UK RCTs. There were four sub-sections in Section A that scored consistently poorly across all leaflets. These sub-sections were: presenting probabilities; clarifying and expressing values; structured guidance in deliberation and communication; and using evidence. This was not surprising given these concepts are not currently included in the informed consent guidance [1,4]. However, the items reflect standards that have been shown to be important to promote high quality decisions for treatment and screening [25] and identify items that have also been shown to be important to trial participants during their trial participation experience [27]. Moreover, our findings are mirrored in a similar study by Brehaut and colleagues, who also demonstrate an absence or lack of detail related to these items in PILs developed for trials in other contexts [34]. Perhaps also unsurprisingly, in our study, scores for Section B were better than scores for Section A. This is perhaps because many of the items included in Section B are present within the current guidelines for informed consent documents [1,3]. Our findings suggest that the information presented in PILs to date has tended to focus on key elements of the RCT rather than supporting the process for decision making. As highlighted earlier, arguably this relates to conceptualisations of trial participation as being merely an act or a process of informed consent. As such, we propose reconsideration of the purpose of PILs and their role in the informed consent process. We would support a move to a more informed decision making process which encourages potential participants to assess what matters to them and how trial participation relates to them personally as individuals. This study provides further evidence that PILs do not include information that has been shown to improve decision making in other contexts. As such, this could suggest that poor quality decisions are being made about trial participation and indicates room for improvement.

There is evidence that some of the items identified as lacking from PILs, which were measured in Section A, may have an important role to play in decision making for participation in RCTs. For example, the section on 'presenting probabilities’ was lacking in most PILs, but aspects of this concept have been shown to be important for decisions about trial participation. In a recent study, parents were faced with a hypothetical decision about inclusion of their child in a RCT of paediatric postoperative pain control. Parents who received probabilistic risk information in pictograph form (a diagram that conveys meaning through pictorial resemblance) understood the information better than those who received information as words or tables [35]. Also, exercises to help patients clarify and express their values, that is, to determine what matters most to them, have been shown to play a potentially important role in decision making for potential RCT participants [30]. A recent study provided potential participants with values clarification exercises when considering participation in a hypothetical trial for breast cancer treatment [30]. These exercises were found to be beneficial in this context as they enabled effective deliberation about the decision by lowering ambivalence and decisional uncertainty and improving the clarity of personal values [30]. Such exercises were also identified as lacking in the PILs in this study. Lastly, another of the areas which scored consistently poorly across the PILs was structured guidance in deliberation and communication. This type of information could be incorporated by including text such as *'*take time to think about how you would feel if you received treatment A rather than treatment B, and how the possible side effects of each would affect you personally’ and 'think carefully about your decision to participate and how it might impact on your life, take time to discuss with family and friends’, and so on. However, the impact and usefulness of such information requires investigation. To our knowledge there is no published research into using structured guidance in deliberation and communication within RCT PILs.

There was no correlation identified between readability and tool score. Interestingly, the average readability level of the PILs in this study (13 to 15 years) is well above the estimated reading age of the UK population in general (9 years) [38]. It is worth noting that readability formulae (such as Flesch-Kincaid) have limitations in that they test 'structure and composition’ rather than meaning or context. In addition they do not take into account additional influences on 'readability’ such as layout, appearance, print size, and use of diagrams [38]. However, there was a significant correlation between word count and tool score. This may be expected as longer PILs can incorporate more information and thus potentially score higher on the evaluation tool. We are not, however, promoting the lengthening of existing PILs, which have previously been noted as getting longer whilst not necessarily improving understanding [39]. It is possible to write shorter PILs whilst improving the overall evaluation score and being in accord with current guidance from ethics committees, as evident from some of the PILs included in this study. For example, PIL01 and PIL17 (word count 1,500 to 2,000) scored higher than others containing substantially more words (PIL04 and PIL15 (word count 2,500 to 3,000)). It may be that the tool should include consideration of word count to accommodate any effect of the number of words in the PIL. Therefore, it may be helpful to develop a framework from the evaluation tool for those writing PILs for RCTs to encourage them to include text that might facilitate better quality decisions which are more aligned with promoting 'informed’ consent.

### Strengths and limitations

The primary strength of this study is that it investigated the content of a sample of UK PILs, considering the extent to which they contained key information which is known to support decision making in other contexts. A further strength of the study is that each PIL was scored by two researchers independently, so as not to introduce rater bias. This study is the first to report the use of Section B of the tool which was developed with the specific aim of assessing consent forms in a US and Canadian context. As discussed earlier, consent forms for US trials are often considerably longer than those in the UK (where they are generally a single page) and much of the information contained within US consent forms would be found in a PIL in the UK. Therefore, we felt it was appropriate to use both sections of the tool to assess UK PILs.

We undertook this study as a feasibility study to assess whether the tool could be used to evaluate PILs from UK-based RCTs, and as such, our sample was small. Although taken from a convenience sample, PILs were sampled purposively to ensure that there was representativeness of CTUs (so as not to introduce any bias from developers) and intervention type across the sample. As we sourced only publicly available PILs from CTU websites, the number of PILs identified is likely to be much smaller than the number of RCTs on-going or recently completed in the UK. Moreover, CTUs may be selective with regard to which PILs they make publicly available on their websites. Despite these potential limitations, we found both variability in scores between PILs and consistency in terms of which sub-sections the PILs scored poorly on. This suggests that our findings may be generalisable, and have relevance to a wider range of PILs for UK RCTs. We restricted our sample to PILs dated from 2001 onwards (to align with the introduction of the European Clinical Trials Directive [36]). Although it is possible that PILs written more recently may score better on the tool, partly because of updates to UK guidance [4], we did not find evidence of an association between year of publication and tool score (r = -0.136, *P* = 0.566). In addition, the majority of the PILs included in our study (16/20) were produced after 2006, and so are likely to conform to the most recent UK guidance documents [4].

### Key recommendations

As is evident from existing studies, changing the structure of information contained within PILs often has little or no effect on knowledge or understanding. However, the findings from this study support new ways of thinking about the information to include in a PIL, which may be more encompassing for supporting high quality decision making, and as such, more aligned with the process of 'informed’ decision making relating to consent. Therefore to improve decision quality with regard to making decisions about trial participation, it may be important to incorporate, or at least consider inclusion of, these items into PILs. The preliminary findings from other studies do offer promise for the use of decision aids, and associated aspects, within the context of informed consent for clinical trials [28-30,35]. We believe there may be potential benefit from the use of decision aids in this context. However, there is currently insufficient evidence to propose a definitive model to improve the existing process such that it aligns more with 'informed decisions’ for trial participation or to recommend writing PILs to IPDAS standards. At present, suggestions for researchers could be to evaluate their own PILs using the tool, equivalent frameworks developed from the IPDAS or explore those items identified in this study as lacking from existing PILs. Any areas which score poorly could be supplemented before the PILs are used in the informed consent process and piloted with potential participants to discuss support for decision making. However, before this could be implemented, empirical studies assessing whether any of these items are associated with an improvement in decision making are required.

It should be recognised that PILs form only one piece of the jigsaw for supporting decisions about trial participation and that decision making in this context is often influenced by much more than just the provided information [27]. We propose that existing PILs need to be replaced, or supplemented, with interventions that are more suited to supporting informed decision making. This may be a decision aid but may also be a strategy for improved communication. It is worth noting that more informed decisions about participation in RCTs may impact either negatively or positively on both recruitment and retention within a study. We postulate that participants who are more fully informed at the outset and more aware of the likely expectations on them throughout their participation experience may be less likely to drop out, but this remains to be determined empirically.

Engagement with key stakeholders such as ethics committees, policy makers, patients and trialists could help to incorporate the findings from this research into the development process for PILs, and pave the way for new ways of thinking about supporting informed decisions in the context of RCT participation.

## Conclusions

We have shown that the evaluation tool can be used to assess whether or not PILs from UK RCTs include items recommended for good quality decision making. Existing PILs for participation in UK RCTs fulfil ethics committee guidelines [1], yet we found that many were lacking in items deemed to be important for promoting high quality decisions. Future research could explore the potential value of the type of information identified as lacking from existing PILs and whether this would better support potential participants to make high quality decisions about participation in RCTs.

## Abbreviations

ANOVA: Analysis of variance; CTU: Clinical Trial Unit; GCP: Good Clinical Practice; ICH: International Conference on Harmonisation; IPDAS: International Patient Decision Aid Standards; NRES: National Research Ethics Service; PIL: Patient information leaflet; RCT: Randomised controlled trial.

## Competing interests

The authors declare that they have no competing interests.

## Authors’ contributions

KG conceived the study, carried out the analysis of PILs, contributed to data analysis and led the writing of the manuscript. WH carried out the analysis of PILs and contributed to data analysis. ZS and SC both participated in the design of the study and coordination and helped to draft the manuscript. JB developed the original evaluation tool. All authors read and approved the final manuscript.

## Supplementary Material

Additional file 1 **Example from coding manual.** The text illustrates an example from the manual that raters used to code patient information leaflets. Click here for file

Additional file 2 **Description of trials included in patient information leaflet sample.** The text illustrates contextual information of patient information leaflets included in the study. Click here for file
