# Supplementary material for: Patient information leaflets (PILs) for UK randomised controlled trials: a feasibility study exploring whether they contain information to support decision making about trial participation
Source: Trials. 2014 Feb 18;15:62. doi: 10.1186/1745-6215-15-62 (PMC3936815; doi:10.1186/1745-6215-15-62)
Supplement: Additional file 1 — Example from coding manual. The text illustrates an example from the manual that raters used to code patient information leaflets. [file 1745-6215-15-62-S1.docx]

**Additional file 1: Example from coding manual**

***Evaluation tool item:*** On the first page of the PIL there is a description that a decision about whether or not to participate in the trial needs to be made.

***Coding rules:***

- Agree: *PIL specifies that a decision needs to be made with reference to trial participation and discusses other options.*
- Disagree: *Does not mention that potential participants need to make that decision (e.g. states ‘you are invited to participate in a study’).*

***Evaluation tool item:*** The PIL describes the disadvantages of participation in the study.

***Coding rules:***

- Agree: *Describe some additional disadvantage of taking part in the trial. e.g. more tests or hospital visits, etc. Risk is not classed as a disadvantage.*
- Disagree: *States there may be disadvantages but with no description.*

***Evaluation tool item:*** The PIL describes important intervention advantages **and/or** disadvantages with a level of detail that helps potential participants imagine the impact on their life**.**

***Coding rules:***

- Agree: *All advantages and/or disadvantages are described to allow the potential participant to imagine how it would make them feel or affect their activities. e.g. Drug X may cause low blood pressure. People with low blood pressure may feel weak or tired.*
- Disagree: *PIL states advantages or disadvantages but with no detail about what it would be like to experience.*
