# Supplementary material for: Patient information leaflets (PILs) for UK randomised controlled trials: a feasibility study exploring whether they contain information to support decision making about trial participation
Source: Trials. 2014 Feb 18;15:62. doi: 10.1186/1745-6215-15-62 (PMC3936815; doi:10.1186/1745-6215-15-62)
Supplement: Additional file 2 — Description of trials included in patient information leaflet sample. The text illustrates contextual information of patient information leaflets included in the study. [file 1745-6215-15-62-S2.docx]

**Additional file 2: Description of trials included in PIL sample.**

| **PIL** | **CTU** | **INTERVENTIONS** | **PRODUCTION DATE** | **SAMPLE SIZE** |
| --- | --- | --- | --- | --- |
| 01 | A | drug *vs*. placebo | 2009 | >1000 |
| 02 | B | drug *vs*. placebo | 2006 | ≤500 |
| 03 | B | drug *vs*. no drug | 2009 | >1000 |
| 04 | C | drug *vs*. no drug | 2008 | * |
| 05 | D | drug *vs*. placebo | 2007 | ≤500 |
| 06 | E | surgery *vs*. placebo surgery | 2002 | ≤500 |
| 07 | F | surgery *vs*. surgery | 2010 | 501-1000 |
| 08 | G | surgery *vs*. surgery + device #1 *vs*. surgery + device #2 | 2009 | >1000 |
| 09 | H | surgery *vs*. surgery | 2006 | >1000 |
| 10 | I | surgery *vs*. surgery + drug | 2005 | ≤500 |
| 11 | J | standard care *vs*. standard care + behavioural intervention #1 *vs*. standard care + behavioural intervention #1 and #2 | 2005 | 501-1000 |
| 12 | K | standard care *vs*. standard care + support intervention | 2010 | * |
| 13 | K | standard care *vs*. cognitive therapy intervention | 2006 | * |
| 14 | L | standard care *vs*. support intervention | 2009 | ≤500 |
| 15 | L | standard care *vs*. support intervention | 2008 | ≤500 |
| 16 | M | standard care *vs*. lifestyle intervention | 2007 | * |
| 17 | N | treatment schedule #1 *vs*. treatment schedule #2  (vary in frequency) | 2005 | * |
| 18 | O | standard care *vs*. device | 2010 | 501-1000 |
| 19 | P | standard care *vs*. standard care +additional information | 2006 | * |
| 20 | Q | standard care *vs*. therapeutic intervention | 2010 | 501-1000 |

*Information not available from PIL.
